# Robust 6-DoF Pose Estimation under Hybrid Constraints

**DOI:** 10.3390/s22228758

**Published:** 2022-11-12

**Authors:** Hong Ren, Lin Lin, Yanjie Wang, Xin Dong

**Affiliations:** 1 Changchun Institute of Optics, Fine Mechanics and Physics, Chinese Academy of Sciences, Changchun 130033, China; 2 University of Chinese Academy of Sciences, Beijing 100049, China; 3 State Key Laboratory on Integrated Optoelectronics, College of Electronic Science and Engineering, Jilin University, Changchun 130012, China

**Keywords:** pose estimation, heatmaps, nonlinear optimization, multi-task networks

## Abstract

To solve the problem of the insufficient accuracy and stability of the two-stage pose estimation algorithm using heatmap in the problem of occluded object pose estimation, a new robust 6-DoF pose estimation algorithm under hybrid constraints is proposed in this paper. First, a new loss function suitable for heatmap regression is formulated to improve the quality of the predicted heatmaps and increase keypoint accuracy in complex scenes. Second, the heatmap regression network is expanded and a translation regression branch is added to constrain the pose further. Finally, a robust pose optimization module is used to fuse the heatmap and translation estimates and improve the pose estimation accuracy. The proposed algorithm achieves ADD(-S) accuracy rates of 93.5% and 46.2% on the LINEMOD dataset and the Occlusion LINEMOD dataset, which are better than other state-of-the-art algorithms. Compared with the conventional two-stage heatmap-based pose estimation algorithms, the mean estimation error is greatly reduced, and the stability of pose estimation is improved. The proposed algorithm can run at a maximum speed of 22 FPS, thus constituting both a performant and efficient method.

## 1. Introduction

The development of image processing technology has allowed computers to extract an increasing amount of information from images. Consequently, object pose estimation at 6 degrees of freedom (DoF) has also become a new research hotspot. This task is proving essential for applications in robotics, autonomous driving, and virtual reality. Therefore, 6-DoF pose estimation has broad application prospects and an extremely high research value.

The high requirements of depth cameras on lighting conditions limit the application scenarios of pose estimation algorithms using RGB-D images. At present, many algorithms for direct pose estimation from RGB images without depth information have achieved relatively good results but the applicability of these algorithms in complex scenes still needs to be improved. In real application scenarios, objects are very often occluded. In these cases, the insufficient information available in the RGB image will limit the accuracy of pose estimation and may even result in serious estimation errors, which limits the usefulness of single RGB images in practical scenarios. Therefore, the maintenance of the accuracy and robustness of the pose estimation algorithm using single RGB images in complex scenes, and especially those with occlusions, is a problem that deserves further study.

Traditional pose estimation algorithms commonly use hand-crafted features to establish an image-to-image relationship and calculate the pose in the real image based on a template image [1,2]. Algorithms of this type are limited by the traditional feature calculation methods and can only deal with objects with a rich texture, but their accuracy advantage has led to many algorithms [3,4,5,6] that adopt the idea of two-stage pose estimation. With the development of deep learning, an increasing number of two-stage pose estimation algorithms are being proposed. These algorithms use the 2D projection of three-dimensional keypoints on the CNN regression model to obtain the image keypoints and then use the PnP method to calculate the pose. There are many methods for regressing image keypoints; among them, heatmaps have been applied in many two-stage pose estimation algorithms because of their accuracy in keypoint regression, their easy supervision and easy convergence during network training, and because they have achieved satisfactory results.

**Figure 1:**
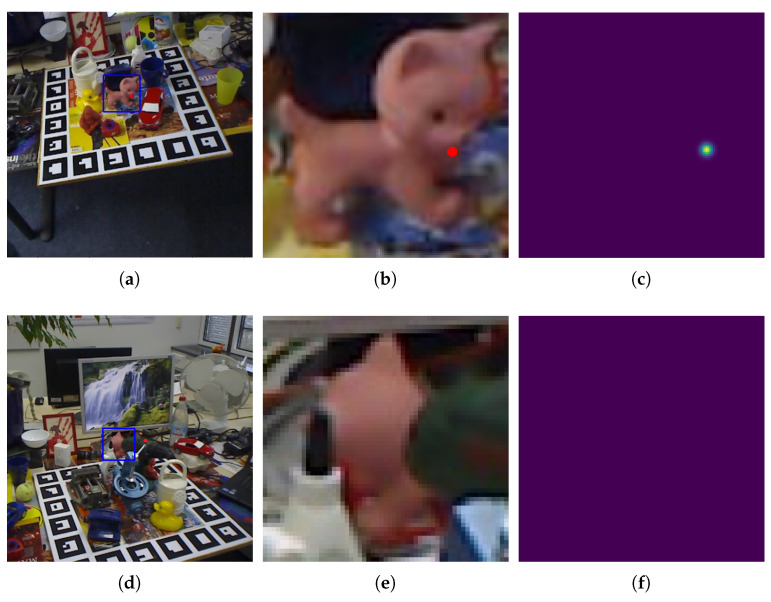
Heatmaps cannot perceive keypoints outside the image. The blue boxes are object bounding boxes and the red points are keypoints. (**a**) Unoccluded image; (**b**) Image block; (**c**) Heatmap for unoccluded keypoints; (**d**) Occluded image; (**e**) Image block; (**f**) Heatmap for occluded keypoints.

**Figure 2:**
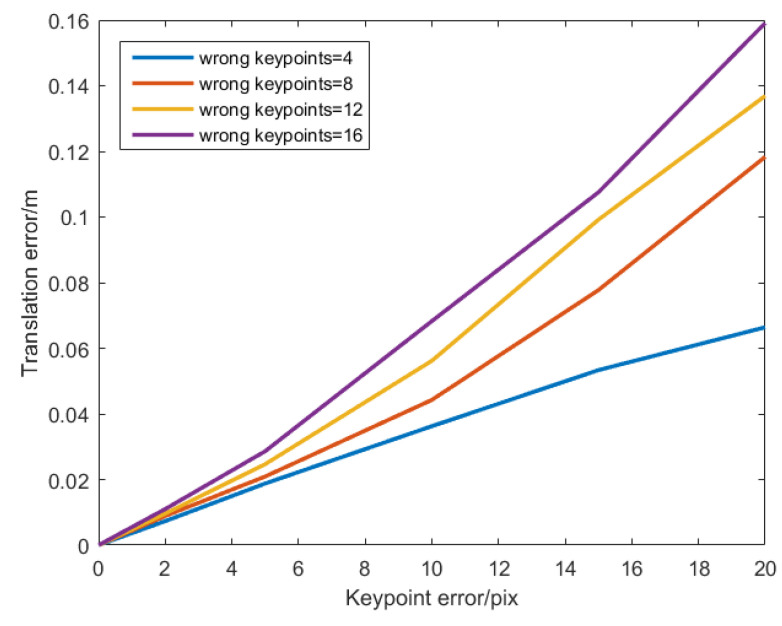
Errors in the determination of the keypoints’ location affects the result of pose estimation.

When using heatmaps, in order to allow the network to use image information more effectively and not be disturbed by the background, object detection and cropping are usually performed, and only image blocks are used for keypoint regression. When the object is occluded, only a part of it is contained in the image block, and some image keypoints may be defined outside the image block. As shown in Figure 1, the heatmap cannot regress keypoints outside the image, which leads to large errors and uncertainties for these keypoints. However, the accuracy of the PnP algorithm in calculating the pose depends almost entirely on the quality of the keypoint regression. As the errors in keypoint calculation increase, the pose calculation results will become more unreliable. Figure 2 shows the result of an image keypoint positioning error affecting pose estimation. The curves in the figure show the translation error caused by errors on different numbers of keypoints when using 20 point pairs to solve the PnP problem. More erroneous keypoints lead to larger translation errors, and this error accumulates almost exponentially. Therefore, when the object is occluded, the two-stage pose estimation algorithm using a heatmap is likely to obtain the pose estimation result with a large error, which reduces the applicability of two-stage pose estimation algorithms using heatmaps on occluded objects.

To solve this problem, some methods [7,8] increase the number of keypoints, so that even when the object is partially occluded, enough high-quality keypoints can be used for the calculation of the pose. However, too many keypoints will increase the difficulty of network training and reduce the computational efficiency. At the same time, it is not easy to distinguish which keypoints should be used to calculate the pose in different images, which results in uncertainty.

In order to improve the accuracy and stability of the two-stage pose estimation algorithm using heatmap on the problem of occluded object pose estimation, this paper improves the pose estimation algorithm using a heatmap from two aspects. First, the prediction of the heatmap by the regression network should be improved, as this will ensure the accuracy of keypoint prediction within the image block range in complex situations. In this regard, in this paper a new loss function called “Heatmap Wing Loss” is proposed, which is used to improve the quality of the predicted heatmap and to ensure the accuracy of keypoint prediction. Second, multiple constraints should be jointly imposed on the pose to ensure the stability of the estimation results. In this paper, inspired by end-to-end algorithms [9,10,11], starting from a heatmap regression network, a translation regression branch is added to predict the translation pose directly. The rotational pose is calculated from the predicted keypoints using the PnP algorithm. At the same time, a robust pose optimization module is proposed, which fuses the two constraints of translation and keypoints to calculate the pose.

The algorithm was evaluated on the LINEMOD dataset and the Occlusion LINEMOD dataset, both of which are widely used standard pose estimation datasets. These two datasets focus on the pose estimation of conventional non-occluded and occluded objects. On both datasets, our algorithm achieves excellent results, outperforming other recent pose estimation algorithms. At the same time, the fastest pose estimation rate of the proposed algorithm is 22 FPS, which makes it suitable for real-time applications.

In summary, in the present work, the two-stage pose estimation algorithm using heatmaps is extended and a new robust pose estimation algorithm under hybrid constraints is proposed. The specific contributions are as follows:

- To improve the accuracy of keypoint positioning, Heatmap Wing Loss is designed specifically for heatmap regression. Using Heatmap Wing Loss, the algorithm improves the quality of the network-predicted heatmap and ensures the stability of keypoint positioning when the object is blocked.
- Referring to the end-to-end algorithm, the heatmap regression network is expanded to add a translation regression branch, so that a variety of constraints on the pose can be imposed, leading to an improvement of the pose estimation stability in occlusion scenes.
- To better integrate the two constraints of the keypoints and translation, a pose optimization module is designed to further improve the accuracy of the estimated poses.

## 2. Related Work

Recently, many excellent algorithms [12,13,14,15] proposed in the BOP Challenge [16,17] aimed to solve the problem of 6D pose estimation using RGB images, and have achieved excellent results in many datasets. However, most of these algorithms are post-refinement algorithms which require a pre-estimation to obtain a rough result in advance. This kind of coarse-to-fine algorithm greatly affects the efficiency and makes it difficult to be applied in real scenarios.

This paper focuses on non-post-optimized pose estimation algorithms using RGB images. These algorithms are mainly divided into two categories. The first is end-to-end methods, which directly predict the pose from the input image through the neural network. The second includes two-stage methods, which obtain the 2D information related to the 3D object points using a neural network, such as when keypoints are projected from 3D model points, and then use the PnP algorithm to solve the pose.

### 2.1. End-to-End Methods

An intuitive approach incorporating deep learning for pose estimation is to use the neural network to output the pose directly. However, due to the large solution space of the pose, the direct regression of translation vectors and rotation matrices is usually inefficient and there are many algorithms that provide different solutions. SSD-6D [10] extends the object detection network SSD to the pose estimation task. It discretizes the output space of the rotation and uses regression to predict the rotation. Deep-6DPose [18] extends the Mask-RCNN [19] network with a pose estimation branch, which uses the 2D object center in the image and the depth of the camera to replace the translation vector, and uses a differentiable Lie algebra for rotation estimation. Li et al. focused on the pose estimation of indoor objects [20], modified Faster R-CNN [21], and added a translation and rotation vector output. The classification and regression methods were used to predict the rotation vector with the conclusion drawn that the regression method has higher accuracy. Different from the method of directly extracting the pose using the image, Martin et al. proposed an Augmented Autoencoders (AAE) [22] by applying template matching. The encoder could automatically obtain the spatial rotation characteristics of the object and the required pose corresponding to a pre-computed template, which greatly simplified the calculation amount. The idea of end-to-end algorithms for pose estimation is simple, but due to the characteristics of the neural network, it is difficult to judge whether the network has learned enough features to express the pose; therefore, the accuracy of these methods is usually poor. However, end-to-end algorithms can be used to refine the pose. For example, DeepIM [23] uses the coarse pose and the 3D model to render the image, and learns the difference between the real image and the rendered image through the neural network, which allows it to calculate the deviation between the coarse pose and the real pose. DeepIM performs pose refinement using PoseCNN and achieves excellent results. However, DeepIM requires a rough estimate as a basis, which includes the real-time performance of the algorithm.

### 2.2. Two-Stage Methods

Compared with end-to-end methods, two-stage algorithms that determine the pose through 2D-3D correspondence have higher accuracy. Two-stage algorithms can be subdivided into direct coordinate methods, heatmap methods, voting methods and dense correspondence methods according to the 2D feature types predicted.

**Direct coordinate methods:** Neural networks can directly predict the coordinates of keypoints from the input image through training. The YOLO-6D algorithm proposed by Tekin et al. [4] draws on the YOLO object detection algorithm, which outputs the projection of the eight corners of the 3D bounding box of the object on the 2D image directly, and proposes a 3D bounding box confidence equation. Kartik Gupta et al. proposed CullNet [24], which changed the original single output of YOLO-6D to three branches, predicted the eight corners at different scales, predicted the corner confidence, and used corners with the highest confidence to calculate the pose. A segmentation-driven pose estimation algorithm was proposed by Hu et al. [25] which segmented the object and predicted the corner positions and confidences of the object’s 3D bounding box within each image block. Finally, the *N* prediction points with the highest confidence are used to apply the PnP calculation of the pose.

Due to the large solution space of directly-predicted coordinates, such methods lack the ability of spatial generalization, and the positioning accuracy of the keypoints is not sufficient, which limits their performance.

**Heatmap methods:** Unlike methods based on direct coordinate prediction, heatmaps provide an intermediate state for coordinate regression, which allows the network to be fully convolutional and consequently easier to train and converge. Heatmaps are widely used to predict keypoints in various pose estimation algorithms. Zhao et al. proposed BetaPose [7], an algorithm that trains a keypoint detector to predict pre-defined keypoints on the object, and began to use heatmaps as an indirect way of expressing keypoints, thereby improving the prediction performance. During training, Zhao et al. [26] used a pair of images as input and used the projection consistency constraint to improve the accuracy of the predicted keypoints on the image. Oberweger et al. [8] proposed an algorithm to deal with occlusion, which divided the object into blocks to ensure that the main body of the input image is not occluded when regressing the keypoints. The use of heatmaps to predict keypoints allows for higher accuracy than direct coordinate prediction; consequently, heatmap methods usually also have better pose estimation performance. However, due to the limitations of the heatmap itself, it cannot predict the keypoints outside the image range, which also affects the application of heatmap-based methods in occluded object pose estimation.

**Voting methods:** To predict keypoints outside the image range, some algorithms use voting-based keypoint localization. Proposed by Yu et al., PoseCNN [9] implements a voting scheme to determine the object center point, regresses the pixel-level unit vector pointing to the center, and then determines the center through multi-pixel voting. Inspired by PoseCNN, Peng et al. proposed the PVNet [27] algorithm, which predicts the direction of all pixels in the object to the keypoints, and uses a RANSAC algorithm-based voting method to determine the keypoints, thus improving the robustness of the pose estimation against occlusion greatly. Yu et al. modified PVNet to use a distance-based loss function, which further improved the speed of network training and the pose estimation performance. HybridPose [28] predicts a hybrid intermediate representation that includes keypoints, edge vectors, and symmetric correspondences, and utilizes different features for accurate pose prediction. ER-Pose [29] upgrades HybridPose’s mask-based voting to edge-based voting, which helps the network to focus more on the object’s global shape and structure. The voting method predicts denser features, and while it can cope with occlusion, it also incurs increased computational cost and thus the inference speed is limited.

**Dense correspondence methods:** In addition to the above algorithms based on sparse pre-defined keypoints, there are also methods based on dense coordinates, which usually predict the pixel-level object’s 3D coordinates or a UV map of pixels to create a dense 2D-3D correspondence, instead of the sparse correspondence found in the above algorithm. These algorithms try to solve the problem that occluded objects are difficult to predict due to pre-defined keypoints that cannot be seen. Kiru Park et al. proposed pix2pose [30], which uses a GAN to predict the 3D model coordinates and confidence levels corresponding to each pixel from the input image directly, and also proposed a new loss function to convert the predicted 3D coordinates of each pixel into its nearest symmetrical pose. DPOD [31], proposed by Zakharov et al., predicts the output UV map from the input image to build pixel-level 2D-3D correspondence. At the same time, DPOD also proposes a refinement module to further improve the accuracy of pose estimation. GDR-Net [32] exploits the intermediate geometric features regarding 2D-3D correspondences to output image-like 2D patches, and 2D convolutional Patch-PnP is used instead of PnP to calculate the pose. The dense coordinate method is robust to severe occlusions; however, due to the large continuous search space, the regression of the coordinates is more difficult than the prediction of sparse keypoints.

Overall, the heatmap method can balance the accuracy of pose estimation and the ease of training and convergence of the network, and is the most likely algorithm to be applied in practice. In order to solve the problem that it is difficult to predict the occluded keypoints in the heatmap method, which leads to difficulty estimating the pose of the occluded object, this paper incorporates the direct prediction of translation. The translation is used together with the heatmap as a constraint to jointly estimate the object pose. Therefore, our method can effectively eliminate the drawbacks of the heatmap method and maintain the stability of pose estimation.

## 3. Proposed Approach

### 3.1. Pipeline

As mentioned above, since heatmaps cannot predict keypoints outside the image, when the object is heavily occluded and there are not enough keypoints included in the image patch, serious errors will occur in the pose estimation. In order to improve the accuracy and robustness of pose estimation, the usual two-stage pose estimation algorithm is extended with the pose estimation algorithm presented in this paper.

**Figure 3:**
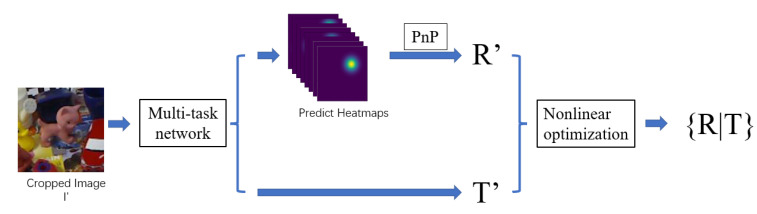
Visualization of proposed pipeline. We use a multi-task network to simultaneously predict the heatmap and translation, and use a nonlinear optimization method to calculate the pose.

In the preliminary stage, object detection is performed on a given image *I* to obtain the bounding box of the object. The image *I* is cropped according to the square bounding box which is calculated using Algorithm 1 to ensure that only the object to be estimated remains in the image block. The cropped image is uniformly scaled to N×N to obtain the image block $I^{\prime }$.
**Algorithm 1** calculation of the output bounding box**Input:** The predicted bounding box of the object, including the upper left coordinate of the bounding box $x_{i},y_{i}$, width $w_{i}$ and height $h_{i}$;**Output:** The square bounding box of the object, including the upper left coordinate of the bounding box $x_{o},y_{o}$, side length $N_{o}$1:Calculate the center of predicted bounding box.
$$\left\{\begin{array}{c} x_{c}=x_{i}+w_{i}/2\\ y_{c}=y_{i}+h_{i}/2 \end{array}\right.$$2:Calculate the side length of the square bounding box.
$N_{o}=1.1*max\left(w_{i},h_{i}\right)$3:Calculate the upper left coordinate of the square bounding box.
$$\left\{\begin{array}{c} x_{o}=x_{c}-N_{o}/2\\ y_{o}=y_{c}-N_{o}/2 \end{array}\right.$$**return**$x_{o},y_{o},N_{o}$;

As shown in Figure 3, our pose estimation algorithm is divided into two stages. In the first stage, the object image block $I^{\prime }$ is input into a multi-task CNN network and the heatmap corresponding to keypoints and the vector $T^{\prime }$ representing the pose translation are the output. The heatmap is then parsed to obtain the predicted image keypoints. After obtaining the predicted image keypoints, the PnP problem is solved to obtain the initial rotation matrix $R^{\prime }$. The second stage uses the initial pose estimate $\left\{R^{\prime }|T^{\prime }\right\}$ as the initial value, and uses a heatmap-based non-linear optimization algorithm to calculate the estimated pose {R|T}.

Each part of the algorithm will be described in detail below.

### 3.2. Mutli-Task Network

**Figure 4:**
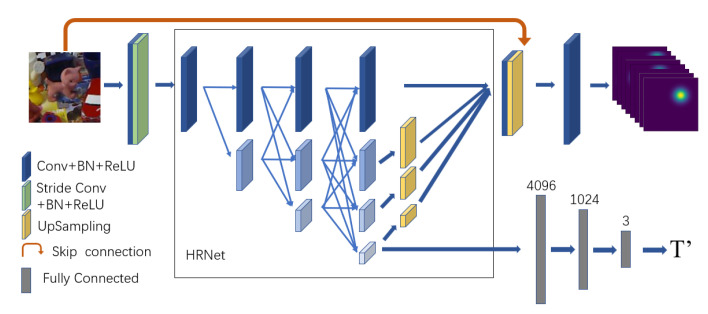
Multi-task network. We extend the heatmap regression structure to output both heatmaps and translations.

Since the heatmap cannot be used to regress keypoints outside the image, its use as the sole constraint to calculate the pose will reduce the stability of the pose estimation. Therefore, it is necessary to increase the constraints of the pose calculation. Different from other heatmap methods such as BetaPose, which only output heatmaps, in this paper the output of the heatmap regression network is expanded to heatmaps corresponding to different keypoints and vectors that represent the object translation pose. The network structure is shown in Figure 4.

In order to reduce the network’s computational load, keypoint prediction algorithms such as BetaPose usually output a heatmap of size N/2×N/2 for an input image of size N×N, and then scale the keypoints to correspond to the input image. The disadvantage of this operation is that there is an error in the keypoints calculated by the prediction heatmap, and the scaling of the keypoints will cause that error to be amplified and accumulated. In the proposed approach, the multi-task network will directly output a heatmap of size N×N to reduce the amplification and accumulation of errors.

In this paper, HRNet [33,34] is selected as the backbone of the multi-task network. HRNet connects the feature maps at different resolutions in parallel, which allows it to retain the high-resolution features during the whole process. In this manner, no features are lost due to the pooling-upsampling operations, which is advantageous for high-accuracy heatmap regression.

To ensure the computational efficiency and reduce the amount of operations, before the image is input into HRNet, a convolutional layer with a stride of 2 is used to reduce the feature map size and improve the inference speed. After fusing the multi-layer features output by HRNet, the fused features are upsampled to the input image size and spliced with the input image. The input image-sized heatmap is obtained after the operation of the last convolution block. When an image of size H×W×3 is used as the input with *C* keypoints defined, the output size of the network heatmap branch is H×W×C.

The prediction of the translation is not very demanding on the features space, and does not require high-resolution feature maps for regression. Moreover, if the high-resolution feature map is used to perform the full-connection calculation directly, the computational workload of the network will be greatly increased, and its computational efficiency will be adversely affected. Therefore, the network expands the lowest resolution feature map output with HRNet in a fully-connected operation. The translation prediction branch uses three fully connected layers with 4096, 1024, and 3, nodes.

Since the image blocks are cropped and scaled before being input to the network, there will be a multi-value correspondence problem between the translation and the image block, so the network cannot be used to predict the translation directly. As an alternative, in this paper the projected position of the object center is regressed in the cropped and scaled image $\left(u_{0},v_{0}\right)$ and the translation z-direction component corresponding to the scaled image block $\hat{t_{z}}$ as the extended translation component.

If the camera internal parameters are

$$\left\{\begin{array}{ccc} f_{x} & 0 & u_{x}\\ 0 & f_{y} & u_{y}\\ 0 & 0 & 1 \end{array}\right\},$$

the cropping position of the image block is $\left(x_{0},y_{0}\right)$, and the cropped image block is enlarged by *k* times to obtain the input image block; then, the relationship between the real translation pose of the object $\left(t_{x},t_{y},t_{z}\right)$ and the output of the network translation branch is as shown in Equation (1). This translation regression is independent of image cropping and scaling.

(1) $$\left\{\begin{array}{ccc} t_{x} & = & \frac{\left(\left(\frac{u_{0}}{k}+x_{0}\right)-u_{x}\right)t_{z}}{f_{x}}\\ t_{y} & = & \frac{\left(\left(\frac{v_{0}}{k}+y_{0}\right)-v_{y}\right)t_{z}}{f_{y}}\\ t_{z} & = & \frac{{\hat{t}}_{z}}{k} \end{array}\right.$$

### 3.3. Loss Function

The loss function of the entire network consists of two parts. First, for the prediction of translation, the L2 Loss is used to evaluate the deviation of the predicted value from the true one. In the extended translation component, $\left(u_{0},v_{0}\right)$ and ${\hat{t}}_{z}$ have different prediction difficulties. Therefore, the loss function of the translation is shown in Equation (2), where $\alpha_{1},\alpha_{2}$ are control parameters, $\left(\bar{u_{0}},\bar{v_{0}},\bar{\hat{t_{z}}}\right)$ and $\left(u_{0},v_{0},\hat{t_{z}}\right)$ are the actual and predicted values of the extended translation vector.

(2) $$Loss_{T}=\alpha_{1}L_{2}\left(\bar{u_{0}},u_{0}\right)+\alpha_{1}L_{2}\left(\bar{v_{0}},v_{0}\right)+\alpha_{2}L_{2}\left(\bar{{\hat{t}}_{z}},{\hat{t}}_{z}\right)$$

In heatmap regression, many algorithms use the mean square error (MSE) loss for training. However, it is not the optimal loss function, as the vast majority of pixels in the heatmap are invalid background zero-valued pixels, and only a few foreground pixels near keypoints have values. When using heatmaps to calculate keypoint location, only a small portion of the foreground pixels with the maximum value and its neighbors are really used. Therefore, different pixel values of the heatmap have different keypoint positioning importance, and the importance of a heatmap pixel increases with its proximity to the keypoint position. However, MSE loss will account for all pixels of the heatmap during the training process, resulting in the reduction in useless background pixel errors for most of the network training time, which is not conducive to the rapid convergence of the network and also affects the prediction accuracy of foreground pixels.

The proposed approach aims to distinguish different pixels in the whole network training process to obtain more accurate prediction results of foreground pixels. Inspired by [35,36], a Heatmap Wing Loss (HWing Loss) function is proposed which is more suitable for heatmap regression. The loss function expression is shown in Equation (3). HWing Loss is designed to be a piecewise function, where *p* and $\hat{p}$ represent the real and predicted pixel value; consequently, the the image pixel error is $p-\hat{p}$. δ, ϵ, and ω are parameters, while θ is the threshold. When the pixel error is greater than θ, the large-error form of the function (second line) is used, which does not distinguish foreground pixels from background pixels and uses a linear function to reduce the impact of outliers on training. When the error is less than θ, the small-error part (first line) is adopted. In this case, due to the characteristics of the ln function, the gradient value of the loss function increases as the error decreases, and the sensitivity to small errors is improved; the exponential part ϵ−p is used to solve the problem of the discontinuous gradient of the ln function when the error is 0. The denominator δ−p allows the loss function to distinguish the foreground from the background pixels, enhances the gradient of the foreground pixel part, and increases the significance of foreground pixels during training.

(3) $$HeatmapWingLoss\left(p,\hat{p}\right)=\left\{\begin{array}{ccc} \omega \ln\left(1+|\frac{p-\hat{p}}{\delta -p}{|}^{\epsilon -p}\right)\; & |p-\hat{p}|<\theta & \\ |p-\hat{p}|-C\; & |p-\hat{p}|>\theta & \end{array}\right.$$

**Figure 5:**
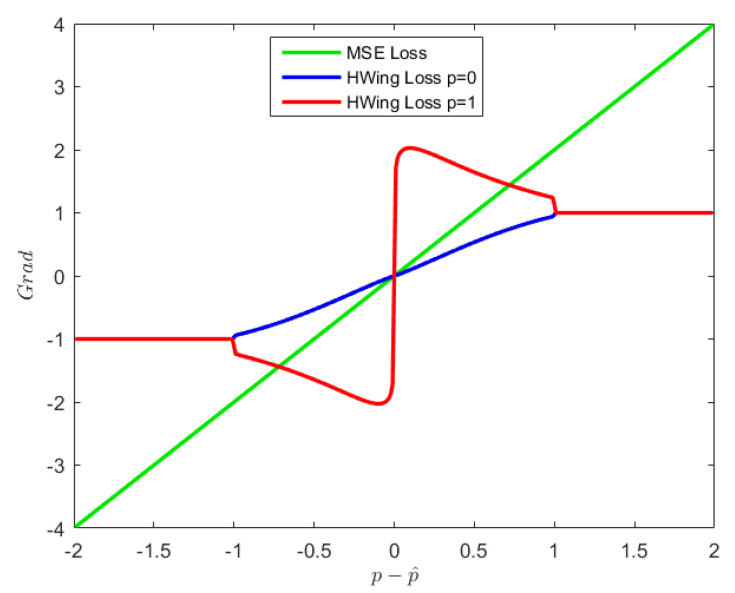
Gradient of Heatmap Wing Loss and MSE Loss.

Figure 5 shows a comparison between HWing Loss and MSE Loss when the background pixel is p=0 and the foreground pixel is p=1. It can be seen that in the small error part, HWing Loss distinguishes better between the gradients of the foreground and background pixels. Smaller pixel errors result in a more obvious distinction, which makes the network pay more attention to and optimize the error of the foreground pixel during the training process. This feature can improve the prediction accuracy of the foreground part, thereby improving the accuracy of keypoint location and pose estimation.

The final overall loss function is shown in Equation (4).

(4) $$\begin{array}{ccc} Loss & = & Loss_{T}+\beta Loss_{heatmap}\\ \; & = & \alpha_{1}L_{2}\left(\bar{u_{0}},u_{0}\right)+\alpha_{1}L_{2}\left(\bar{v_{0}},v_{0}\right)+\alpha_{2}L_{2}\left(\bar{\hat{t_{z}}},\hat{t_{z}}\right)+\beta \sum_{N}\sum_{p}HWingLoss\left(p,\hat{p}\right) \end{array}$$

### 3.4. Initial Pose

After the network output has been obtained, the initial pose $\left\{R^{\prime }|T^{\prime }\right\}$ can be calculated. The initial translation $T^{\prime }$ can be obtained using Equation (1), while the keypoints are used for the calculation of the initial rotation $R^{\prime }$.

In order to obtain more accurate keypoints from the heatmaps, the algorithm in [37] was expanded to perform sub-pixel keypoint optimization, as shown in Equation (5), where (u,v) are the optimized keypoint coordinates, $\left(u_{0},v_{0}\right)$ are the coordinates of the maximum point of the heatmap, and $L_{u}^{\prime }$, $L_{v}^{\prime }$, $L_{u}^{\prime \prime }$, and $L_{v}^{\prime \prime }$ represent the first- and second-order differences at the $\left(u_{0},v_{0}\right)$ position. After obtaining the keypoints, the maximum pixel values of each heatmap are used to filter the keypoints. The $K_{1}$ points with the largest maximum values are selected to calculate the initial pose. The initial rotation $R^{\prime }$ is calculated using the EPnP algorithm [38].

(5) $$\left\{\begin{array}{ccc} u & = & u_{0}-\frac{L_{u}^{\prime }\left(u_{0}\right)}{L_{u}^{\prime \prime }\left(u_{0}\right)}\\ v & = & v_{0}-\frac{L_{v}^{\prime }\left(v_{0}\right)}{L_{v}^{\prime \prime }\left(v_{0}\right)} \end{array}\right.$$

### 3.5. Pose Optimization

The initial pose $\left\{R^{\prime }|T^{\prime }\right\}$ obtained in Section 3.4 is not sufficiently accurate for high-precision applications. The reason is that when using the keypoints to calculate the initial pose, although the keypoints have been screened using the maximum heatmap values, the number of erroneous keypoints in different instances may not be the same, and the EPnP algorithm cannot reduce the influence of the wrong key points during calculation. Therefore, the initial pose can be further optimized using nonlinear optimization.

When the position of the keypoints obtained by the network regression is correct, then any 3D keypoint $P_{oi}$ should coincide with the predicted image keypoint $p_{i}$ after mapping to the image after applying the rotation *R*, the translation *T* and internal parameter *K*. Since the predicted values of rotation *R* and translation *T* are inaccurate, for each keypoint there is the following error:

(6) $$e_{i0}={∥p_{i}-\left(KRP_{oi}+T\right)∥}^{2}$$

However, there are also errors in the position of the keypoints predicted by the network. The position of $p_{i}$ itself is inaccurate. Image keypoints with large errors will lead to large errors in pose calculation or even wrong estimations. It is therefore necessary to reduce the influence of inaccurate keypoints. Considering that the keypoints are inferred from the predicted heatmap, the accuracy of keypoints is often correlated with the quality of that heatmap. Therefore, the keypoint error can be determined using the maximum value $m_{i}$ of the predicted heatmap. The closer the maximum value $m_{i}$ of the predicted heatmap is to 1, the higher the quality of the heatmap, and consequently the lower the error of the keypoint. On the contrary, if the maximum value $m_{i}$ of the predicted heat map is closer to 0, this indicates that the quality of the heatmap is poor, which may be caused by occlusion or keypoints outside the image range, and the larger the keypoint error at this time. After combining the heatmap quality, the error corresponding to each keypoint is as follows:

(7) $$e_{i}=m_{i}e_{i0}={∥m_{i}\left(p_{i}-\left(KRP_{oi}+T\right)\right)∥}^{2}≜d_{i}^{2}$$

Similar to Section 3.4, the $K_{2}$ keypoints with the largest predicted heatmap maxima are selected for pose optimization, where $K_{2}$ is less than or equal to $K_{1}$ in Section 3.4. To further reduce the influence of outliers on optimization and improve the robustness of nonlinear optimization, we define the total error of the $K_{2}$ keypoints as shown in Equation (8), where *c* is a parameter.

(8) $$E=\sum_{i=1}^{K_{2}}\frac{c^{2}}{2}\log\left(1+{\left(\frac{d_{i}}{c}\right)}^{2}\right)$$

(9) $$\frac{\partial }{\partial d_{i}}E=\frac{d_{i}}{1+{\left(\frac{d_{i}}{c}\right)}^{2}}$$

Equation (9) provides the partial derivative of the total error *E* to each keypoint error $d_{i}$. Compared with using $\sum e_{i}$ directly, the error definition of Equation (8) can significantly reduce the proportion of outlier errors in the total error, as well as the influence of outliers on optimization. Smaller values of the parameter *c* result in a smaller gradient of the error function and consequently the influence of outliers on optimization is suppressed. However, if *c* is too small, the correct prediction points with small errors will be mistaken as outliers, which will affect the correctness of optimization. Therefore, it is necessary to choose the value of parameter *c* reasonably.

Finally, the Levenberg–Marquardt (-L–M) algorithm is used to optimize Equation (10) and achieve pose optimization.

(10) $$\begin{array}{ccc} R*,T* & = & \underset{R,T}{\arg\min}E\\ \; & = & \underset{R,T}{\arg\min}\sum_{i=1}^{K_{2}}\frac{c^{2}}{2}\log\left(1+{\left(\frac{m_{i}\left(p_{i}-\left(KRP_{oi}+T\right)\right)}{c}\right)}^{2}\right) \end{array}$$

## 4. Experiments

### 4.1. Benchmark Datasets

We trained and evaluated the algorithm using the LINEMOD dataset [39] and the Occlusion LINEMOD dataset [40].

The LINEMOD dataset is commonly used for 6-DoF object pose estimation. There are multiple pose estimation scenes in the dataset, including complex backgrounds and non-textured objects. Almost all objects in the scenes are not occluded. Each image has an object and is annotated with its translation, rotation, object class, and object mask. The dataset also provides 3D models. There is a total of 15,783 images of 13 categories, including 11 asymmetrical categories, namely ape, benchvise, cam, can, cat, driller, duck, holepuncher, iron, lamp, phone, as well as 2 symmetrical categories, namely eggbox and glue. There are approximately 1200 instances of each of the categories.

The Occlusion LINEMOD dataset is an extension of the LINEMOD dataset which only includes 8 categories, namely ape, can, cat, driller, duck, eggbox, glue, and holepuncher. There are multiple objects marked with poses, categories, and masks in each image and most objects in the image are partially occluded. The Occlusion LINEMOD dataset was used for evaluation only, and only the LINEMOD dataset is used for training.

Usually, when using the LINEMOD dataset, 30% of the images in each category are randomly selected as the training set and the remaining 70% is used as the test set. As a result, the amount of data in the training set is too small, which results in the network having insufficient accuracy and being prone to overfitting. Therefore, the strategy of [27] was used to add synthetic images to the training set, resulting in a total of 20,000 training images per class. Among these image, 10,000 images are rendered whose viewpoints are uniformly sampled, while another 10,000 images are synthesized using the “Cut and Paste” strategy.

### 4.2. Evaluation Metrics

The algorithm’s performance was evaluated using the ADD(-S) metric, which considers the mean distance between the 3D points of the model. For asymmetric objects, the ADD metric is used, and the mean distance is the mean distance between the coordinates of the vertices of the 3D model and the estimated coordinates, as shown in Equation (11), where $N_{p}$ is the total number of 3D points on the 3D model, and $x_{i}$ is the *i*-th 3D point. If the mean distance is less than 10% of the object’s diameter, the prediction is considered to be correct. For symmetric objects, the ADD-S metric is used, and the mean distance is calculated based on the nearest point distance, as shown in Equation (12). The performance evaluation is calculated as the percentage of the number of correctly predicted images in the test set of the total number.

(11) $$ADD\left(R,T\right)=\frac{1}{N_{p}}\sum_{i=1}^{N_{p}}\left(\left(Rx_{i}+T\right)-\left(\bar{R}x_{i}+\bar{T}\right)\right)$$

(12) $$ADD-S\left(R,T\right)=\frac{1}{N_{p}}\sum_{i=1}^{N_{p}}\min_{x_{j}}\left(\left(Rx_{i}+T\right)-\left(\bar{R}x_{j}+\bar{T}\right)\right)$$

### 4.3. Implementation

The farthest point sampling algorithm was used to select C=32 points on the 3D model as 3D keypoints, map them to the image to obtain image keypoints and subsequently create supervised heatmaps. The input images were uniformly cropped and scaled to 128×128 pixels. Each batch comprised 32 images. The learning rate was set to 0.001. All models were trained for 40 epochs and the learning rate was reduced to 0.0001 from the 30th epoch onwards. In Heatmap Wing Loss, the parameters were set to ω=14, θ=0.5, δ=2, and ϵ=2.1. In the overall loss function, $\alpha_{1}=1$, $\alpha_{2}=5$, and β=1 were used. For the evaluation, $K_{1}=K_{2}=24$ keypoints with the largest confidence coefficient were selected for initial pose estimation and optimization. The pose optimization module was implemented using Ceres [41].

### 4.4. Ablation Studies

**Figure 6:**
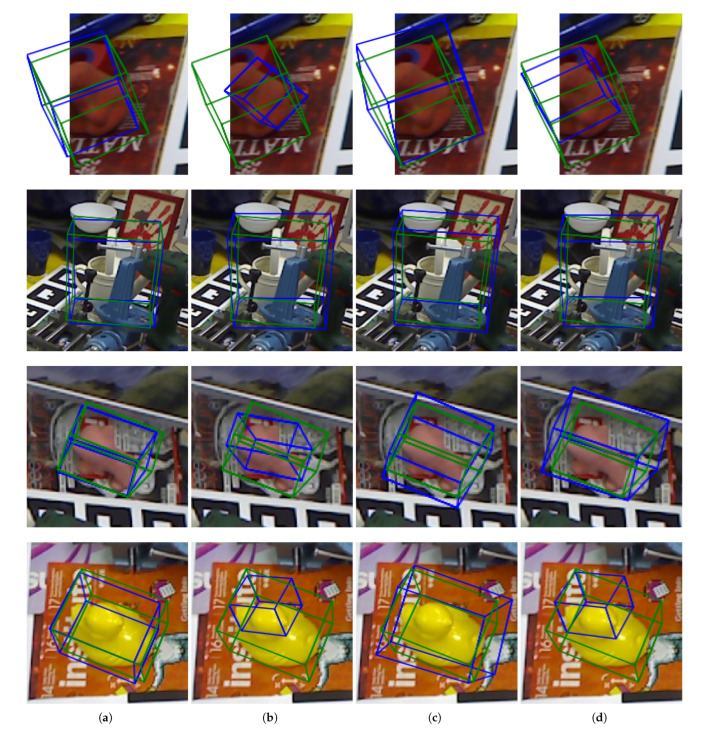
Visualized results of different algorithm configurations. (**a**): full algorithm; (**b**): without the optimization module; (**c**): without translation model; (**d**): without optimization module and translation module.

In order to verify the role and performance of the translation and optimization modules, ablation studies were conducted on the Occlusion LINEMOD dataset. The performance of the algorithm with and without the translation and optimization modules was compared to that of the original algorithm, which included both. The results are shown in Table 1, which compares the ADD(-S)/% accuracy and ADD(-S)/m error of the different algorithms. Figure 6 presents the visualization results of some examples.

From the results, it can be seen that the basic algorithm without the use of translation and optimization modules achieves an accuracy of 41.2% on the Occlusion LINEMOD dataset. The use of the optimization module results in an accuracy increase of 5.3 percentage points (pp). The optimization effect is clear, indicating that the nonlinear optimization module is robust to outliers, as shown in the third column and fourth column of Figure 6.

The accuracy of the algorithm using the translation module alone on the Occlusion LIMEMOD dataset is significantly lower than that of the basic algorithm. However, the accuracy of the algorithm when both the translation and the optimization modules are used is almost the same as that of the algorithm using only the optimization module, which further illustrates the improvement of the optimization module to the accuracy of the algorithm’s pose estimation.

The effect of the translation module is reflected in the ADD(-S)/m error term, which represents the mean difference measured using the ADD distance between the real pose and the estimated pose of all data in the test set. The ADD(-S)/m error is shown in Equation (13), where ADD(-S)(R,T) is defined in Equations (11) and (12), and $N_{data}$ is the total number of data in the test set. Compared with using only the optimization module, employing both modules results in a reduction in the ADD(-S) error by more than 75%. When the translation module is not used, the difficult categories are significantly misestimated, resulting in a high ADD(-S) error. This is the case especially for cat, eggbox, and glue categories, where the ADD(-S) error exceeded 1m. However, when the translation module was employed, the ADD(-S) error of most categories was reduced to less than 0.4 m, and the effect was very obvious. This shows that the addition of the translation module improves the robustness of the algorithm’s pose estimation and reduces erroneous estimation.

(13) $$ADD\left(-S\right)/m=\frac{\sum^{N_{data}}ADD\left(-S\right)\left(R,T\right)}{N_{data}}$$

The reason why the translation module improves the stability of pose estimation is that when the algorithm only uses one keypoint constraint but the number of accurate keypoints is too small, such as less than 4, too many wrong keypoints will cause large errors in the final pose, which will result in large translation errors in the estimated poses of some instances. After adding the direct translation prediction, a more accurate initial value is used for the pose calculation, which constraints the calculation and reduces the influence of the erroneous keypoints on the estimated result. Therefore, there is no excessively large error estimation for each instance, and the stability of pose estimation for difficult-to-estimate objects is maintained.

In this study, comparative experiments with different loss functions were carried out to verify the effectiveness of HWing Loss. In order to exclude the influence of other modules, comparative experiments were carried out using the basic pose estimation algorithm without the translation and optimization modules. In the experiment, MSE Loss and HWing Loss were used to train the heatmap regression network and tested on the Occlusion LINEMOD dataset. Table 2 shows the mean keypoint localization errors (in pixel units) of the pose estimation algorithm trained with different loss functions on the Occlusion LINEMOD dataset. The algorithm trained with HWing Loss has an average reduced keypoint location error of 2.2 pixels in each category compared to the one trained using MSE lossMore precise keypoint positioning bestows the pose estimation algorithm trained with HWing Loss with higher pose estimation accuracy.

**Figure 7:**
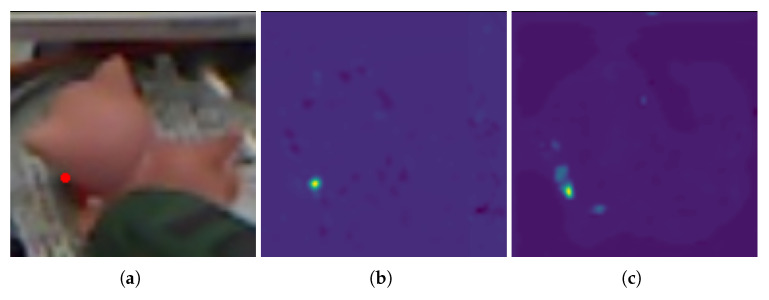
Predicted heatmap using HWing Loss and MSE Loss. (**a**) occluded object and keypoint; (**b**) heatmap using HWing Loss; (**c**) heatmap using MSE Loss.

Figure 7 shows a comparison of heatmaps predicted by a heatmap regression network trained with HWing Loss and MSE Loss. It can be seen that the effective foreground part of the predicted heatmap obtained using MSE Loss is more scattered, while HWing Loss results in a heat map foreground part that is more concentrated. Higher quality prediction heatmaps also result in more accurate keypoint predictions.

### 4.5. Performance on LINEMOD

**Figure 8:**
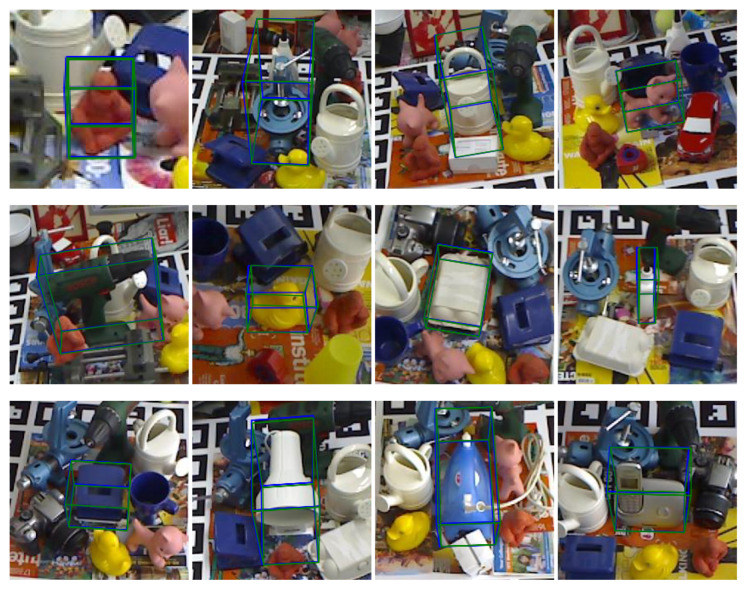
Visualizations of results on the LINEMOD dataset.

We evaluated the performance of the algorithm for unoccluded object pose estimation on the LINEMOD dataset. Figure 8 depicts some qualitative results, where green is the ground-truth and blue is the estimated pose annotated frame.

To verify the performance of the proposed algorithm, the performance of ADD(-S) on the LINEMOD dataset was compared with other current pose estimation algorithms using RGB images. The specific results are shown in Table 3, where the highest accuracy rate of a category is shown in bold. Since eggbox and glue are symmetrical objects, the ADD-S indicator was used. Regarding the compared algorithms, BetaPose [7], CDPN [42], DPVL [43], RPVNet [44], HybridPose [28], ER-Pose [29], and GDR-Net [32] are two-stage methods like the proposed one. BetaPose [7] is the same as ours in terms of using a heatmap, CDPN [42], and GDR-Net [32] use dense correspondence, and DPVL [43], RPVNet [44], HybridPose [28], and ER-Pose [29] are voting methods. Meanwhile, PoseCNN [9] is an end-to-end algorithm, and DeepIM [23] is a refinement-based algorithm that relies on the refinement of PoseCNN’s rough estimation.

From the comparison results, is is evident that among all non-post-refinement algorithms, the proposed algorithm has the highest accuracy rate in four categories, and has the highest mean pose estimation accuracy. Compared with BetaPose, which is also a heatmap method, the proposed algorithm greatly improves the estimation accuracy (the accuracy rate is increased by 20.9 pp). Compared with DeepIM, which uses the rough pose of PoseCNN and then applies a CNN module for refinement, the proposed algorithm also achieves an accuracy improvement of 4.9 pp. The results indicate that the proposed algorithm achieves excellent pose estimation performance compared to these state-of-the-art algorithms.

### 4.6. Performance on Occlusion LINEMOD

**Figure 9:**
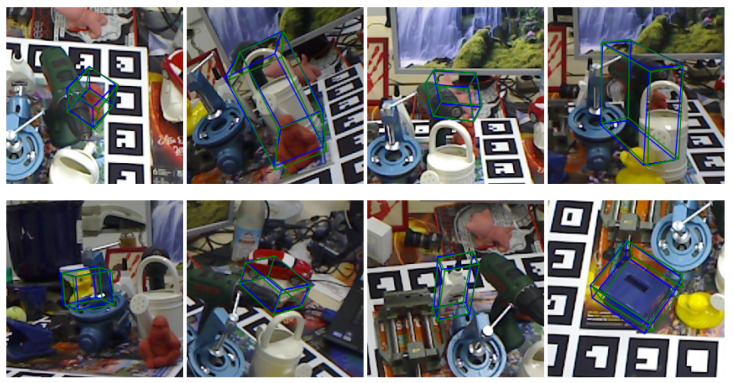
Visualizations of results on the Occlusion LINEMOD dataset.

To evaluate the proposed algorithm’s performance on occluded object pose estimation, the algorithm was applied on the Occlusion LINEMOD dataset. Some qualitative results are shown in Figure 9, where the real pose annotation is shown in green and the estimated pose annotation is blue. It can be seen that the proposed algorithm can maintain excellent pose estimation accuracy even when the object is partially occluded or at extreme angles.

A performance comparison using the ADD(-S) metric was also conducted between the proposed and the other pose estimation algorithms using RGB images on the Occlusion LINEMOD dataset. The results are shown in Table 4, where bold designates the the highest accuracy rate of a category. The ADD-S metric was used for the egg box and glue category objects due to their symmetry. For the compared algorithms, HeatmapNet [8] is a heatmap method which applies image segmentation to improve the quality of the heatmap regression of occluded keypoint; DPVL [43], RPVNet [44], HybridPose [28], and ER-Pose [29] use the voting method, DPOD [31] and GDR-Net [32] employ dense correspondence, while DPOD+Ref adds a CNN-based refinement module on the basis of DPOD.

From the comparison results, it is evident that the proposed algorithm has the highest mean pose estimation accuracy among algorithms without post-refinement. Among the algorithms with known class data, our algorithm has the leading accuracy rate in five classes. HeatmapNet [8] uses image segmentation and is optimized for occluded objects, but it still lags behind the proposed algorithm by 15.8 pp. The voting and dense correspondence methods predict dense features. Compared with our algorithm, they theoretically have natural advantages in estimating the pose of the occluded object. However, in terms of results, our algorithm has a similar pose estimation accuracy with voting and dense correspondence methods, which is better than DPVL [43], and RPVNet [44], while the accuracy is slightly worse than HybridPose and ER-Pose. The refined version of the DPOD is only 1 pp better than the proposed one, which also demonstrates the superior performance of the proposed algorithm in the problem of occluded object pose estimation. It is worth mentioning that compared with the voting method or dense correspondence method used in other algorithms, our algorithm uses a more simple and direct sparse correspondence method. Therefore, our algorithm has only been trained for 40 epochs, while other algorithms have been trained for at least 200 epochs. In other words, our algorithm can achieve equally excellent results using fewer resources. Overall, our method is found to be one of the leading RGB-based pose estimation algorithms.

### 4.7. Running Time

Our algorithm is based on object detection; therefore, the running time depends on the object detection network performance. For a 640×480 image, when using an Intel I7-9700K CPU and NVIDIA RTX3080 GPU, the object detection time using yolo-v5 was about 11 ms. The multi-task network forward inference took about 23 ms; the heatmap inference keypoint location required about 5 ms; the EPnP algorithm ran for about 0.3 ms in order to solve the initial pose; and the nonlinear optimization required about 5 ms. The overall pose estimation took about 45 ms, corresponding to a rate of about 22 frames per second, which means that the proposed algorithm is suitable for real-time pose estimation.

## 5. Discussion

In this paper, by proposing a new heatmap loss function and increasing the pose estimation constraint, a new two-stage pose estimation algorithm using heatmap is provided, which improves the accuracy and stability of the algorithm in the pose estimation problem of occluded objects. However, there is still room for improvement.

1. In this paper, only the translation component is used for the pose constraint. In addition, the predicted rotation component, keypoint correlation information and symmetry information can all be used to constrain the pose. Using more constraints on pose in the algorithm is more conducive to improving the stability of pose estimation.
2. It is difficult for the two-stage algorithm using heatmap to estimate the pose of occluded object. The main reason is that the heatmap cannot locate key points outside the range of the image. Therefore, we can learn from the idea of voting method to generate features that represent key points outside the image range within the image range.
3. This paper only focuses on the pose estimation problem of a single object in an image, when there are multiple objects in the image, the algorithm needs to estimate the pose of each object one by one, which affects the operation efficiency of the algorithm. How to estimate multiple objects in an image at the same time will be studied later.

## 6. Conclusions

In this paper, a robust pose estimation algorithm is proposed under hybrid constraints for accurate and robust estimation of the translation and rotation of an object relative to the camera. Our pose estimation algorithm can predict the heatmap and the translation simultaneously. Heatmap Wing Loss is proposed in heatmap regression to improve the accuracy of keypoint prediction on occluded objects. The addition of translation estimation provides a relatively accurate constraint to the pose when the object is occluded and the keypoint prediction is inaccurate, thus improving the robustness of the pose estimation algorithms on occluded objects. At the same time, a robust pose optimization method is used to fuse the heatmaps and translation estimate, which further improves the pose estimation accuracy. The proposed algorithm achieved state-of-the-art results on both the LINEMOD and the Occlusion LINEMOD datasets. In the future, how to further improve the accuracy of pose estimation of occluded objects will be studied.

## Figures and Tables

**Table 1:** Comparison results of module ablation studies.

Metric	ADD(-S)/%	ADD(-S)/m	ADD(-S)/%	ADD(-S)/m	ADD(-S) /%	ADD(-S)/m	ADD(-S)/%	ADD(-S)/m
**Trans. Module**	✓		✓		✕		✕	
**Opt. Module**	✓		✕		✓		✕	
ape	31.5	0.14	14.8	0.08	32.3	0.33	25.8	0.41
can	75.1	0.04	10	0.06	75.8	0.03	69.8	0.03
cat	30.6	0.66	4.9	0.17	26.8	3.02	24	2.79
duck	34.6	0.35	11.3	0.09	31.1	0.76	28.3	0.65
driller	56	0.09	29	0.06	61.7	0.09	53.4	0.10
eggbox	46.7	0.38	1.4	0.13	51.4	1.11	42.1	1.13
glue	53.4	0.36	21.6	0.10	51.1	3.72	50.5	6.25
holepuncher	42	0.03	8	0.05	41.9	0.03	35.6	0.03
mean	46.2	0.26	12.6	0.09	46.5	1.14	41.2	1.42

**Table 2:** Comparison of mean keypoint location errors on the Occlusion LINEMOD dataset of algorithms trained with different loss functions.

	Mean Keypoint Location Error/cm	
**Loss Function**	**Heatmap Wing Loss**	**MSE Loss**
ape	8.4	9.1
can	4.1	4.6
cat	13	15.1
duck	9	10
driller	5.2	6.8
egg box	38.4	39.2
glue	14.6	20.7
hole	8.2	12.8
Mean	12.6	14.8

**Table 3:** Comparison of methods on the LINEMOD dataset in terms of the ADD(-S) metric.

Method	Proposed	BetaPose	CDPN	DPVL	RPVNet	HybridPose	ER-Pose	poseCNN	DeepIM
ape	73	41.2	64.4	69.1	55.6	63.1	62.6	-	**77**
benchvise	99.5	85.7	97.8	**100**	98.7	99.9	**100**	-	97.5
cam	**96.9**	78.9	91.7	94.1	83.6	90.4	95.8	-	93.5
can	**99.3**	85.2	95.9	98.5	93.2	98.5	99.2	-	96.5
cat	**92.3**	73.9	83.8	83.1	75.5	89.4	90.7	-	82.1
driller	98	77	96.2	**99**	94.7	98.5	**99**	-	95
duck	72.7	42.7	66.8	63.5	63.5	65	68.6	-	**77.7**
eggbox	99.8	78.9	99.7	**100**	95.8	**100**	**100**	-	97.1
glue	96.6	72.5	**99.6**	98	93.4	98.8	98.7	-	99.4
hole.	**95.3**	63.9	85.8	88.2	82.5	89.7	89.7	-	52.8
iron	98.1	94.4	97.9	99.9	96.1	**100**	99.6	-	98.3
lamp	99.4	98.1	97.9	**99.8**	96.8	99.5	99.4	-	97.5
phone	95	51	90.8	96.4	91.5	94.9	**96.8**	-	87.7
Mean	**93.5**	72.6	89.9	91.5	86.1	91.3	92.3	62.7	88.6

**Table 4:** Method accuracy comparison on the Occlusion LINEMOD dataset in terms of the ADD(-S) metric.

Method	Proposed	HeatmapNet	RPVNet	DPVL	DPOD	HybridPose	ER-Pose	GDR-Net	DPOD+Ref
ape	31.5	17.6	17.9	19.2	-	20.9	25.9	39.3	-
can	75.1	53.9	69.5	69.8	-	75.3	72.1	79.3	-
cat	30.6	3.3	19	21.1	-	24.9	25.3	23.5	-
duck	34.6	19.2	31.1	34.3	-	27.9	35.8	44.4	-
driller	56	62.4	63.7	71.6	-	70.2	72.9	71.3	-
eggbox	46.7	25.9	59.2	47.3	-	52.4	48.7	58.2	-
glue	53.4	39.6	46.6	39.7	-	53.8	58.8	49.3	-
hole.	42	21.3	42.8	45.3	-	54.2	47.4	58.7	-
Mean	46.2	30.4	43.7	43.5	32.8	47.5	48.3	53.0	47.2

## Data Availability

The data used to support the findings of this study are available from the corresponding author upon request.
